# *Chákṣu*: A glaucoma specific fundus image database

**DOI:** 10.1038/s41597-023-01943-4

**Published:** 2023-02-03

**Authors:** J. R. Harish Kumar, Chandra Sekhar Seelamantula, J. H. Gagan, Yogish S. Kamath, Neetha I. R. Kuzhuppilly, U. Vivekanand, Preeti Gupta, Shilpa Patil

**Affiliations:** 1 Department of Electrical and Electronics Engineering, Manipal Institute of Technology Manipal, Manipal Academy of Higher Education, Manipal, 576104 India; 2 Department of Electrical Engineering, Indian Institute of Science, Bangalore, 560012 India; 3 Department of Ophthalmology, Kasturba Medical College Manipal, Manipal Academy of Higher Education, Manipal, 576104 India; 4 Department of Ophthalmology, ASRAM, Eluru, 534005 India; 5 Hind Institute of Medical Sciences, Sitapur, 261303 India; 6 Aastha Superspeciality Eye Hospital, Banashankari, Bangalore, 560085 India

**Keywords:** Optic nerve diseases, Eye abnormalities

## Abstract

We introduce *Chákṣu*–a retinal fundus image database for the evaluation of computer-assisted glaucoma prescreening techniques. The database contains 1345 color fundus images acquired using three brands of commercially available fundus cameras. Each image is provided with the outlines for the optic disc (OD) and optic cup (OC) using smooth closed contours and a decision of normal versus glaucomatous by five expert ophthalmologists. In addition, segmentation ground-truths of the OD and OC are provided by fusing the expert annotations using the mean, median, majority, and Simultaneous Truth and Performance Level Estimation (STAPLE) algorithm. The performance indices show that the ground-truth agreement with the experts is the best with STAPLE algorithm, followed by majority, median, and mean. The vertical, horizontal, and area cup-to-disc ratios are provided based on the expert annotations. Image-wise glaucoma decisions are also provided based on majority voting among the experts. *Chákṣu* is the largest Indian-ethnicity-specific fundus image database with expert annotations and would aid in the development of artificial intelligence based glaucoma diagnostics.

## Abstract (summary)

**Table:** 

Measurement(s)	glaucoma severity grading
Technology Type(s)	cup-to-disc ratio
Factor Type(s)	Indian ethnicity fundus image database for glaucoma prescreening
Sample Characteristic - Organism	Human
Sample Characteristic - Environment	Ophthalmology
Sample Characteristic - Location	India

## Background & Summary

Glaucoma is a chronic, irreversible, and slowly progressing optical neuropathy that damages the optic nerve^1,2^. Depending on the extent of damage to the optic nerve, glaucoma can cause moderate to severe vision loss. Glaucoma is asymptomatic in the early stages. It is not curable, and the lost vision cannot be restored. However, by early screening and detection, the progression of the disease could be slowed down. Color fundus imaging is the most viable non-invasive means of examining the retina for glaucoma^2^. The widest application of fundus imaging is in optic nerve head or optic disc examination for glaucoma management. Fundus imaging is widely used due to the relative ease of establishing a digital baseline for assessing the progression of the disease and the effectiveness of the treatment. Fundus imaging technology is developing rapidly and several exciting products with fully automated software applications for retinal disease diagnosis are on the horizon^2–5^. State-of-the-art tools based on image processing and deep learning algorithms are becoming increasingly useful and relevant. However, before deploying them in a clinical setting, a thorough validation over benchmark datasets is essential. The development of a large database with multiple expert annotations is a laborious and tedious task. A large annotated glaucoma-specific fundus image database is lacking, which is a gap that the *Chákṣu* database reported in this paper attempts to fill. Several retinal fundus image databases are publicly available to facilitate research and performance comparison of segmentation and classification algorithms. The salient features of various databases are explained in the following and also highlighted in Table 1.

**Table 1:** Comparison of *Chákṣu IMAGE* with benchmark fundus image databases.

Database	# Cameras/FoV	Image resolution in pixels	# Images	Objective	Ground-truth labels
ARIA^14^	One/50	768 × 576	143	Age-related macular degeneration assessment	OD, retina vessels, fovea center
DRIONS-DB^15^	One/−	600 × 400	110	OD segmentation	OD boundary
Drishti-GS^9,11^	One/30	2896 × 1944	101	Glaucoma classification	OD and OC boundary, CDR values, glaucoma decision
IDRiD^7,8^	One/50	4288 × 2848	516	Diabetic retinopathy analysis	Hard and soft exudates, microaneurysms, hemorrhages, OD boundary
LES-AV^16^	One/60	1622 × 1444	22	Glaucoma classification Vessel analysis	Glaucoma decision, Retinal vessels
Messidor^17,18^	One/45	2304 × 1536, 2240 × 1488, 1440 × 960	1200	OD analysis	OD boundary, fovea center
ONHSD^19,20^	One/45	640 × 480	99	OD segmentation	OD boundary
ORIGA^21^	−/−	—	650	Glaucoma classification	OD and OC boundary, glaucoma decision
REFUGE^12,13^	Two/45	2124 × 2056, 1634 × 1634	1200	Glaucoma classification	OD and OC boundary, glaucoma decision, fovea center
RIGA^23^	−/−	2304 × 1536, 2240 × 1488, 1440 × 960, 2743 × 1936, 2376 × 1584	750	OD analysis	OD and OC boundary
RIM-ONE^24^	One/45	2144 × 1424	169	Glaucoma classification	OD boundary, glaucoma decision
STARE^25,26^	One/35	605 × 700	400	OD localization	OD location
***Chákṣu IMAGE*** **(Ours)**	**Three**/**40**	**2448** × **3264, 2048** × **1536, 1920** × **1440**	**1345**	**OD and OC segmentation Glaucoma classification**	**OD and OC boundary, glaucoma decision**

The symbol “—” indicates ‘information not reported’ by the authors.

Databases such as the one available with Kaggle^6^ (provided by EyePACS) and Indian Diabetic Retinopathy Image Dataset (IDRiD)^7,8^ are part of image analysis competitions for diabetic retinopathy (DR) detection. Drishti-GS^9–11^ and Retinal Fundus Glaucoma Challenge (REFUGE)^12,13^ are glaucoma-specific databases and provide expert annotations of both OD and OC boundaries and binary decisions on glaucoma. REFUGE was the first and largest publicly available glaucoma-specific database (1200 images) with OD and OC ground-truth annotations for 800 images and glaucoma binary decisions for 400 images.

Automatic Retinal Image Analysis (ARIA) database^14^ contains 143 color fundus images of size 768 × 576. ARIA provides OD segmentation and blood vessel masks created by trained experts and also annotation of the fovea center. OC segmentation mask and glaucoma decisions are not available.

The Digital Retinal Images for Optic Nerve Segmentation Database (DRIONS-DB)^15^ has 110 fundus images. The image resolution is 600 × 400 pixels and is accompanied by OD ground truth contours from two experts. The average age of the subjects is 53 years, all of them belonging to Caucasian ethnicity, with the gender distribution of the subjects being 54% female and 46% male. About 23% of the patients had chronic glaucoma and 77% ocular hypertension.

Drishti-GS database^9,11^ consists of 101 fundus images of the Indian population. Each image has a resolution of 2896 × 1944 pixels. The dataset is divided into train and test subsets. The training subset has 50 images with OD and OC segmentation ground truths and notching information. The test set has 51 images for which the ground truth is available. The subjects were in the range of 40–80 years with a nearly equal number of females and males. Ground truth was collected from four experts with varying clinical experience of 3, 5, 9, and 20 years. The database provides OD and OC segmentation soft-maps fused on one binary image, average OD and OC boundaries, and cup-to-disc ratio (CDR) values from four expert markings. It also provides image-level normal or glaucomatous decisions based on the majority opinion (3 out of 4) of experts and a decision on the occurrence of notching in the superior, inferior, nasal, and temporal sectors assessed by a single expert.

The IDRiD database^7,8^ contains 516 fundus images and has a mixture of disease stratification representatives of diabetic macular edema and diabetic retinopathy (DR). The images have a resolution of 4288 × 2848 pixels. The dataset provides expert DR lesion marking and normal retinal structures. The severity level of DR and diabetic macular edema are provided for each image based on internationally accepted and clinically relevant standards. The OD segmentation ground truth, OD and fovea center locations are also provided. This dataset also contains 81 fundus images with signs of DR. Precise pixel-level annotations of microaneurysms, soft exudates, hard exudates, and hemorrhages are provided as binary masks.

The LES-AV dataset^16^ comprises 22 fundus images with corresponding manual annotations for the blood vessels, marked as arteries and veins. The images include labels for glaucomatous and healthy conditions.

Les Méthodes d Evaluation de Systèmes de’Segmentation et d’Indexation Dédiées à l’Ophtalmologie Rétinienne (Messidor)^17,18^ stands for methods to evaluate segmentation and indexing techniques in the field of retinal ophthalmology. The Messidor database contains 1200 colour fundus images with resolutions of 2304 × 1536, 2240 × 1488, and 1440 × 960 pixels. 800 images were acquired with pupil dilation and 400 without dilation. The 1200 images are made available in three subsets of 400 images each. The database provides OD ground truth and fovea center annotation by a single clinician.

The Optic Nerve Head Segmentation Dataset (ONHSD)^19,20^ contains 99 fundus images taken from 50 patients. The subjects are from various ethnic backgrounds (Asian - 20%, Afro-Caribbean - 16%, Caucasian - 50%, Unknown - 14%). The images are of 640 × 480 resolution. The OD outline is marked by four clinicians.

The Online Retinal Fundus Image Database for Glaucoma Analysis and Research (ORIGA) database^21^ contains 650 images with OD and OC segmentation and glaucoma severity grading information. However, the database is not publicly available.

The REFUGE database^12,13^ consists of 1200 images acquired from subjects of Chinese ethnicity using two devices–a Zeiss Visucam 500 fundus camera with a resolution of 2124 × 2056 pixels (400 images); and a Canon CR-2 device with a resolution of 1634 × 1634 pixels (800 images). Each image in the database includes a normal/glaucomatous label. 90% of the database (1080 images) corresponds to normal subjects, while the remaining 10% (120 images) corresponds to glaucomatous subjects. The database is divided into three subsets: training, offline, and online test sets. The training set contains higher-resolution images acquired with Zeiss Visucam 500 camera, while the offline and online test sets include the lower-resolution images captured with Canon CR-2 device. OD and OC manual annotations using tilted ellipses were provided by seven independent glaucoma specialists with an average experience of 8 years. The ground-truth for each image was obtained by a majority voting of the expert annotations. Information pertaining to localization of Fovea (the center of the macula) is provided for 400 images. The second version of the Retinal Fundus Glaucoma Challenge (REFUGE2) was organized in the year 2020^22^ with the objective of evaluating and comparing automated algorithms for OD and OC segmentation and glaucoma detection. REFUGE2 dataset has 800 new fundus images on top of 1200 images from REFUGE.

Retinal fundus images for glaucoma analysis (RIGA) dataset^23^ contains 750 fundus images with OD and OC segmentation ground truth but there are no labels indicating glaucoma severity.

The Retinal Image Database for Optic Nerve Evaluation (RIM-ONE) database^24^ contains 169 images, of which, 118 are classified as normal, 12 as early glaucoma, 14 as moderate glaucoma, 14 as deep glaucoma, and 11 as ocular hypertension.

The Structured Analysis of Retina (STARE) database^25,26^ consists of 400 fundus images acquired using a Topcon TRV-50 fundus camera with a resolution of 605 × 700 pixels. Out of the 400 images, 81 have OD localization ground-truth, and are comprised of 31 images of healthy retinas and 50 images of retinas with a disease.

**Fig. 1:**
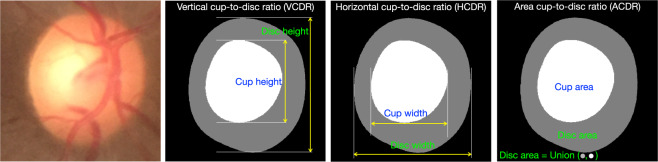
Vertical CDR (VCDR), horizontal CDR (HCDR), and area CDR (ACDR) from the segmented OD and OC.

The review of the existing databases shows that only Drishti-GS^9,11^ and REFUGE^12,13,22^ databases provide both OD and OC segmentation ground-truth and glaucoma decisions along with a clear split of training and testing sets. Drishti-GS provides fused OD and OC segmentation ground-truth using annotations from four experts. However, the individual expert OD and OC segmentation ground-truths are not provided. REFUGE provides a ground-truth by a majority voting across seven experts who provided the manual annotation, but doesn’t provide the individual expert annotations. Further, OD and OC ground-truth are marked only on 800 images (400 in training set and 400 in validation set) using an oriented ellipse. The shape-specific outline is a potential source of bias in the computation of parameters relevant to glaucoma. Ground-truth outlines that rely on elliptical templates would naturally be biased in favor of ellipse-fitting algorithms. ARIA^14^, DRIONS-DB^15^, IDRiD^7,8^, ONHSD^19,20^, and Messidor^17,18^ provide OD segmentation outlines/ground-truth. However, OC segmentation ground-truth and glaucoma decisions are not provided. LES-AV^16^ provides glaucoma decisions, but not OD and OC segmentation ground-truth. RIGA^23^ provides OD and OC segmentation ground-truth but not glaucoma decisions. RIM-ONE^24^ provides OD ground truth and glaucoma decisions but not OC segmentation ground truth. ORIGA^21^ is not yet publicly available. STARE^25,26^ provides only OD localization ground-truth. None of the publicly available fundus image databases provide the OD height/width/area, OC height/width/area, and neuroretinal rim, which are crucial in the computation of clinically relevant glaucoma parameters such as vertical cup-to-disc ratio (VCDR), which is the ratio of vertical height of OC to OD; horizontal cup-to-disc ratio (HCDR), which is the ratio of horizontal width of OC and OD; and area cup-to-disc ratio (ACDR), which is the ratio of areas of OC and OD from the expert annotations (cf. Fig. 1). There is a lack of a sizeable glaucoma-specific database with multi-expert annotations and ground-truths.

## Methods

We introduce a new database namely *Chákṣu IMAGE* to aid the evaluation of computer-assisted glaucoma prescreening techniques for OD and OC segmentation, computation of VCDR, HCDR, and ACDR, glaucoma decisions, etc. The word *Chákṣu* refers to the *eye* in Sanskrit. Minor variations of this word, all referring to the *eye*, exist in several Indo-European languages. *IMAGE* is an acronym for **I**ISc-**MA**HE **G**laucoma **E**valuation database. This database is the result of an interdisciplinary collaboration between the Indian Institute of Science (IISc), Bangalore and Manipal Academy of Higher Education (MAHE), Manipal, India.

### Subject recruitment and image acquisition

The subjects were recruited at the out-patient department (OPD) of the Department of Ophthalmology, Kasturba Medical College (KMC), Manipal and at various departments of Manipal Institute of Technology (MIT), Manipal Academy of Higher Education (MAHE), Manipal, Karnataka, India after obtaining necessary approvals from MAHE Institutional Review Board and Ethics Committee. The study adhered to the tenets of the Declaration of Helsinki^27^. The subjects are in the age group of 18 to 76 years and gave informed consent for data acquisition. The subjects are of Indian ethnicity–a demography that has not been covered adequately in the state-of-the-art fundus image databases. The subjects underwent an undilated fundus examination by an experienced ophthalmologist with the support of a technician as part of the standard clinical workflow. The data collection drive was carried out over a period of two years.

The database consists of 1345 retinal color fundus images acquired using three brands of commercially available fundus imaging devices. The images acquired are 32-bit RGB and stored in JPEG/PNG format. The images acquired are approximately OD-centered and the acquisition devices used are: Remidio^28^ non-mydriatic Fundus-on-phone (FoP) camera with a resolution of 2448 × 3264 pixels (1074 images), Forus 3Nethra Classic^29^ non-mydriatic fundus camera with a resolution of 2048 × 1536 pixels (126 images), and a Bosch^30^ handheld fundus camera with a resolution of 1920 × 1440 pixels (145 images). The patient’s personal information was anonymized. The database of 1345 fundus images is divided into training and test subsets comprising 1009 images and 336 images, respectively, approximately in the ratio of 3:1.

### Expert annotations

**Fig. 2:**
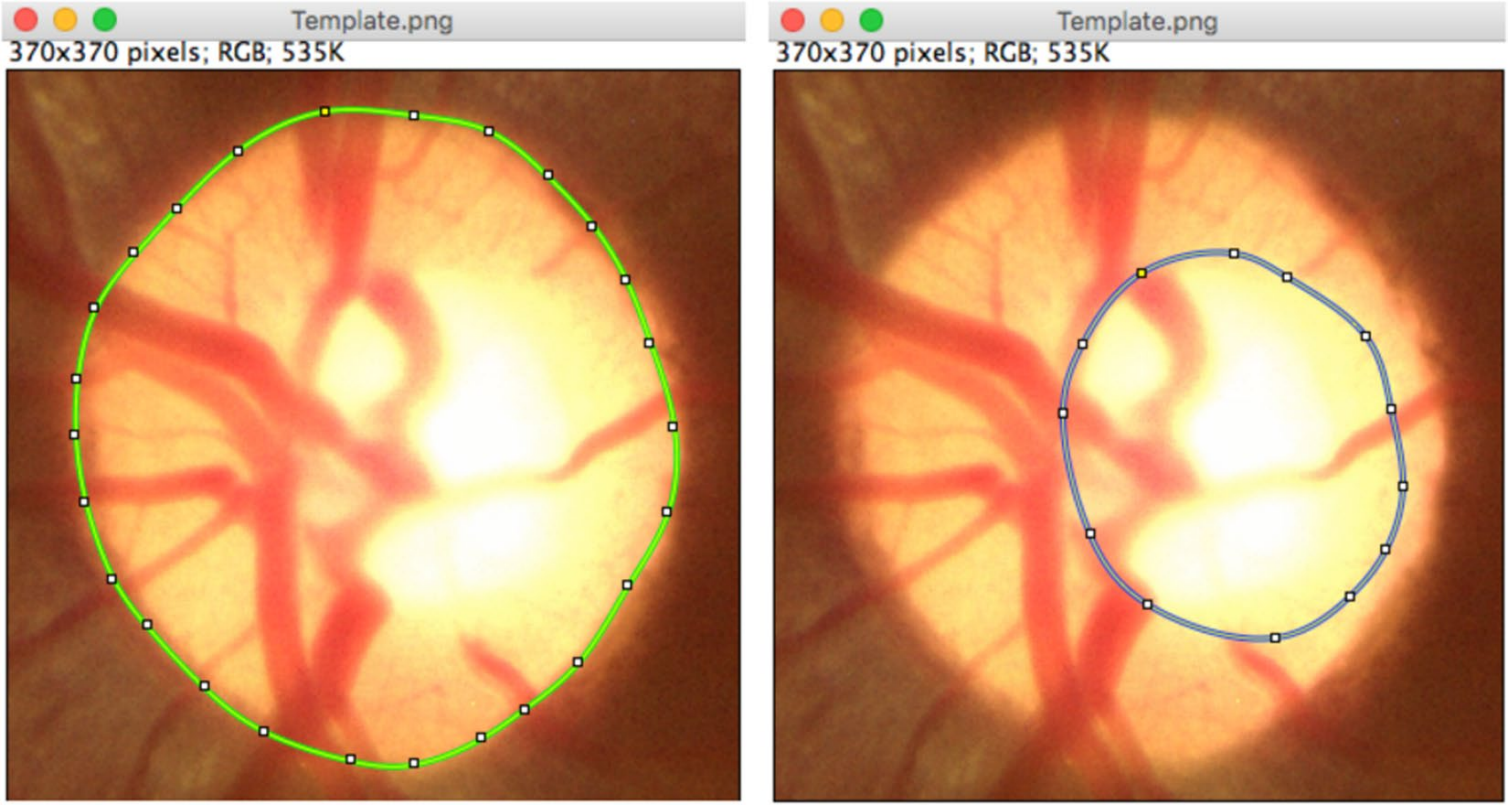
Examples of optic disc, and optic cup annotation provided by an expert using the ImageJ annotation tool.

**Fig. 3:**
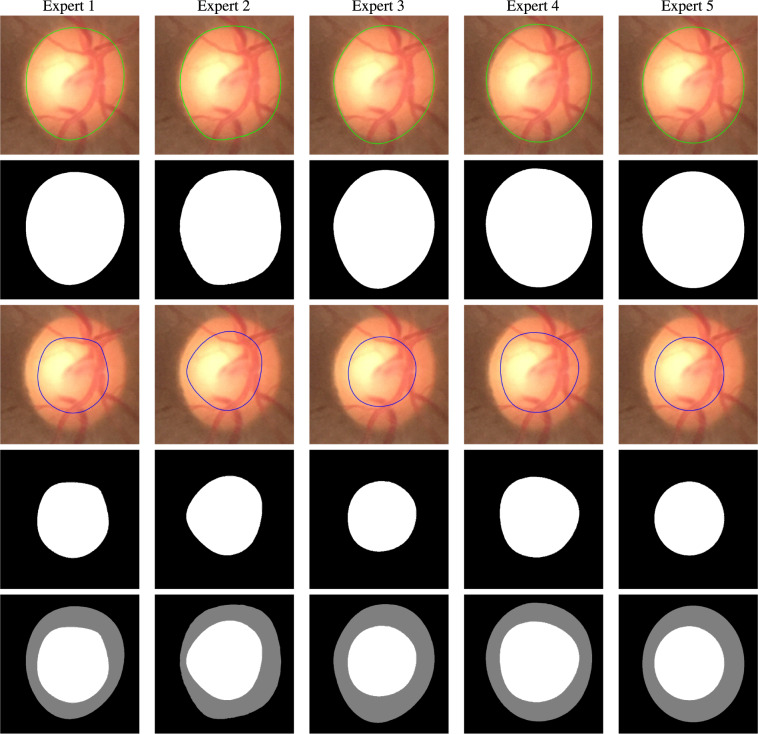
OD (Row 1) and OC (Row 3) segmentation by experts, and their binary representations (Row 2 and Row 4, respectively). Row 5 shows the combined OD and OC annotations.

Five expert Indian ophthalmologists provided the OD and OC segmentation ground-truth and a binary decision on whether the subject is glaucomatous or not. Two of the experts are experienced Professors, two Associate Professors, and one is a clinical practitioner. Three of the experts are glaucoma specialists and two are general ophthalmologists, with experience ranging from 5 to 15 years. They are also coauthors of this paper. In order to overcome bias due to shape-specific (for instance, the tilted-ellipse) OD and OC annotation in some of the existing databases, our experts used smooth closed contours for manual delineation of the OD and OC. The annotation tool is based on ImageJ^31,32^, which is a widely used Java-based image processing program developed at the National Institutes of Health, USA. The experts specify several knot points (greater than 10) on the boundary of the OD/OC using ImageJ’s *polygon selection* tool. The points are connected using cubic B-spline interpolation. We used the cubic B-spline kernel as it possesses the minimum-curvature interpolation property^33^. The experts were given the flexibility to edit the knot locations, update the contour, and save the final outline. An illustration of the contours is provided in Fig. 2, wherein the OD and OC are shown in green and blue contours, respectively. The contours are used to arrive at a binary decision mask, which serves as the ground-truth for the OD and OC segmentation. Figure 3 shows the OD and OC outlines provided by the experts on a cropped fundus image together with their binary representations and fusion of expert OD and OC segmentation. In addition to the outline, the experts also provide binary glaucomatous/nonglaucomatous decisions, which are decided by a majority vote to arrive at a single decision per image.

Table 2 summarizes the key features of the database.

**Table 2:** Features of the *Chákṣu IMAGE* database.

Particulars	Training set	Test set
Total number of images (1345)	1009	336
Fundus imaging devices used and number of images acquired per device:		
Remidio (nonmydriatic type; image resolution: 2448 × 3264)	810	264
Bosch (nonmydriatic type; image resolution: 1920 × 1440)	104	41
Forus (nonmydriatic type; image resolution: 2048 × 1536)	95	31
Number of experts participated in manual annotations	5	5
OD/OC outlining provided by each expert	✓	✓
Single mean OD/OC ground truth generated out of 5 manual annotations	✓	✓
Single median OD/OC ground truth generated out of 5 manual annotations	✓	✓
Single majority OD/OC ground truth generated out of 5 manual annotations	✓	✓
Single STAPLE algorithm based OD/OC ground truth generated out of 5 manual annotations	✓	✓
Parameters computed from expert annotations:	✓	✓
OD, OC, and neuroretinal rim area	✓	✓
Vertical cup-to-disc ratio (VCDR)	✓	✓
Horizontal cup-to-disc ratio (HCDR)	✓	✓
Area cup-to-disc ratio (ACDR)	✓	✓
Glaucoma decision provided by each expert	✓	✓
Single glaucoma decision generated by a majority vote	✓	✓

### OD and OC segmentation ground-truth

**Fig. 4:**
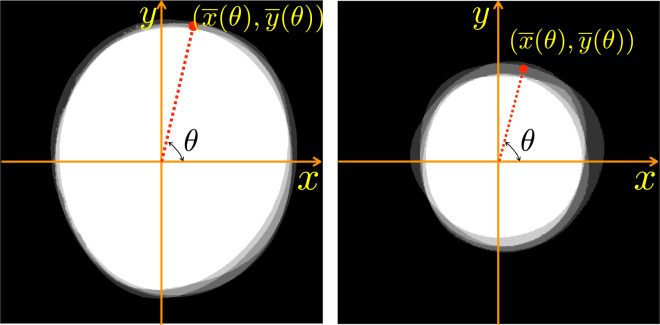
Median ground-truth computation using the *x* and *y* coordinates of the expert outlines as a function of the polar angle *θ*.

**Fig. 5:**
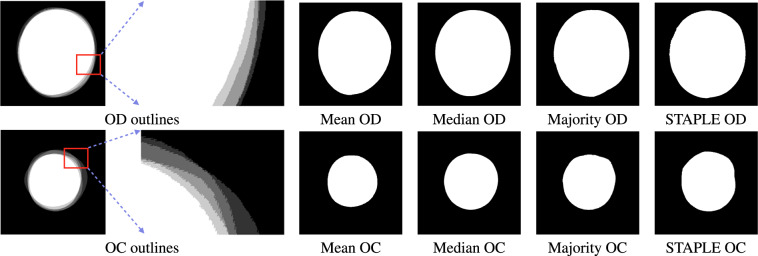
Fusion of binary annotations of experts’ OD (from Row 2 of Fig. 3) and OC (from Row 4 of Fig. 3) segmentation along with the mean, median, majority, and STAPLE ground-truths.

Annotation by several experts is essential to account for inter-expert variability. However, to quantify the performance of a technique, it would be useful to have a single ground-truth. We consider the fusion of the expert annotations based on the mean, median, majority, and STAPLE algorithms. The mean segmentation ground-truth is the region agreed upon by all the experts and is determined by the intersection of all five annotations. We also propose a novel median-based fusion technique. Consider the parametrization of the *x* and *y* coordinates of the OD and OC outlines in terms of the polar angle $\theta \in [0,2\pi )$ as shown in Fig. 4. We compute the median coordinates $\bar{x}(\theta )$ and $\bar{y}(\theta )$ as follows: $\bar{x}(\theta )={\rm{median}}\left({x}_{1}(\theta ),{x}_{2}(\theta ),{x}_{3}(\theta ),{x}_{4}(\theta ),{x}_{5}(\theta )\right)$; and $\bar{y}(\theta )={\rm{median}}\left({y}_{1}(\theta ),{y}_{2}(\theta ),{y}_{3}(\theta ),{y}_{4}(\theta ),{y}_{5}(\theta )\right),$ where *x*_*i*_(*θ*) and *y*_*i*_(*θ*) denote the *x* and *y* coordinates, respectively, at angle *θ*, of the annotation given by Expert *i*. The closed contour formed by $\bar{x}(\theta )$ and $\bar{y}(\theta )$ represents the median ground-truth boundary. The median is proposed as a reliable fusion technique as it is robust to outliers. The majority ground-truth is obtained as the union of the regions agreed upon by at least three experts out of five. Finally, the STAPLE algorithm^34^ based ground-truth boundary is also obtained. STAPLE stands for “Simultaneous Truth and Performance Level Estimation” and is an iterative weighted voting algorithm. It is widely used in the validation of medical image segmentation algorithms due to its robustness and high accuracy. The fusion of the OD and OC segmentations together with various fused ground-truth outlines is illustrated in Fig. 5. One could leverage the multi-expert annotations and several fused ground-truths to perform data augmentation for training machine learning algorithms.

## Data Records

*Chákṣu IMAGE*^35^ is available at the public figshare repository: 10.6084/m9.figshare.20123135 and has the following directory/file structure:

The folder 1.0_Original_Fundus_Images in the Train set contains 104, 95, and 810 color fundus images acquired using Bosch, Forus, and Remidio devices, respectively. The folder 1.0_Original_Fundus_Images in the Test set contains 41, 31, and 264 color fundus images acquired using Bosch, Forus, and Remidio devices, respectively. The folder 2.0_Doctors_Annotations in the Train/Test set contains the expert annotations of OD and OC. The binary segmentation of OD and OC is contained in the folder 3.0_Doctors_Annotations_binary_OD_OC in the Train/Test set. The folder 4.0_OD_OC_Fusion_Images in the Train/Test set contains binary images of OD and OC fused into one. The folder 5.0_OD_OC_Mean_Median_Majority_STAPLE in the Train/Test set contains the overlay, mean, median, majority, and STAPLE algorithm-based binary ground-truths. The folder 6.0_Glaucoma_Decision in the Train/Test set contains glaucoma decisions of the experts and also a majority voting-based decision.

## Technical Validation

For analyzing the experts’ annotations and ground-truths, we consider a subset of the training set containing 810 fundus images captured by the Remidio device. The mean, median, majority, and STAPLE ground-truth OD and OC images were derived from the expert annotations. Sensitivity (*S*_*e*_), specificity (*S*_*p*_), accuracy (*A*_*c*_), error (*E*_*p*_), Jaccard (*J*), and Dice (*D*) similarity indices are standard objective measures that are used to quantify image segmentation performance^36,37^. These indices are computed based on the true positives (*TP*), false positives (*FP*), false negatives (*FN*), and true negatives (*TN*). Sensitivity (or true positive rate) is defined as the proportion of positives that are correctly identified as positives. Specificity (or true negative rate) is defined as the proportion of negatives that are correctly identified as negatives. Accuracy measures the degree of closeness of algorithm segmentation to that of the expert. The performance indices are computed as shown below:

$${\rm{Sensitivity}}\,({S}_{e})=\frac{TP}{\left(TP+FN\right)},$$

$${\rm{Specificity}}\,({S}_{p})=\frac{TN}{\left(TN+FP\right)},$$

$${\rm{Accuracy}}\,({A}_{c})=\frac{\left(TP+TN\right)}{\left(TP+TN+FP+FN\right)},{\rm{and}}$$

$${\rm{Error}}\,({E}_{r})=(1-{A}_{c}).$$

Sensitivity could be unity for a poor segmentation much larger than the ground truth. Specificity is therefore the necessary counterpart of Sensitivity, but it could equal one even for a poor segmentation that does not detect the region of interest. Jaccard similarity index (*J*) is the ratio between the intersection and union. Dice similarity index (*D*) is closely related to the Jaccard similarity index and one could be deduced from the other. They are given as follows:

$${\rm{Jaccard}}\,\mathrm{index}\,(J)=\frac{| A\cap M| }{| A\cup M| },$$

$${\rm{Dice}}\,\mathrm{index}\,(D)=\frac{2| A\cap M| }{| A| +| M| },$$

where *A* and *M* represent the region of interest segmented by the algorithm and the medical expert, respectively. By definition, 0 ≤ *J* ≤ 1 and 0 ≤ *D* ≤ 1.

**Fig. 6:**
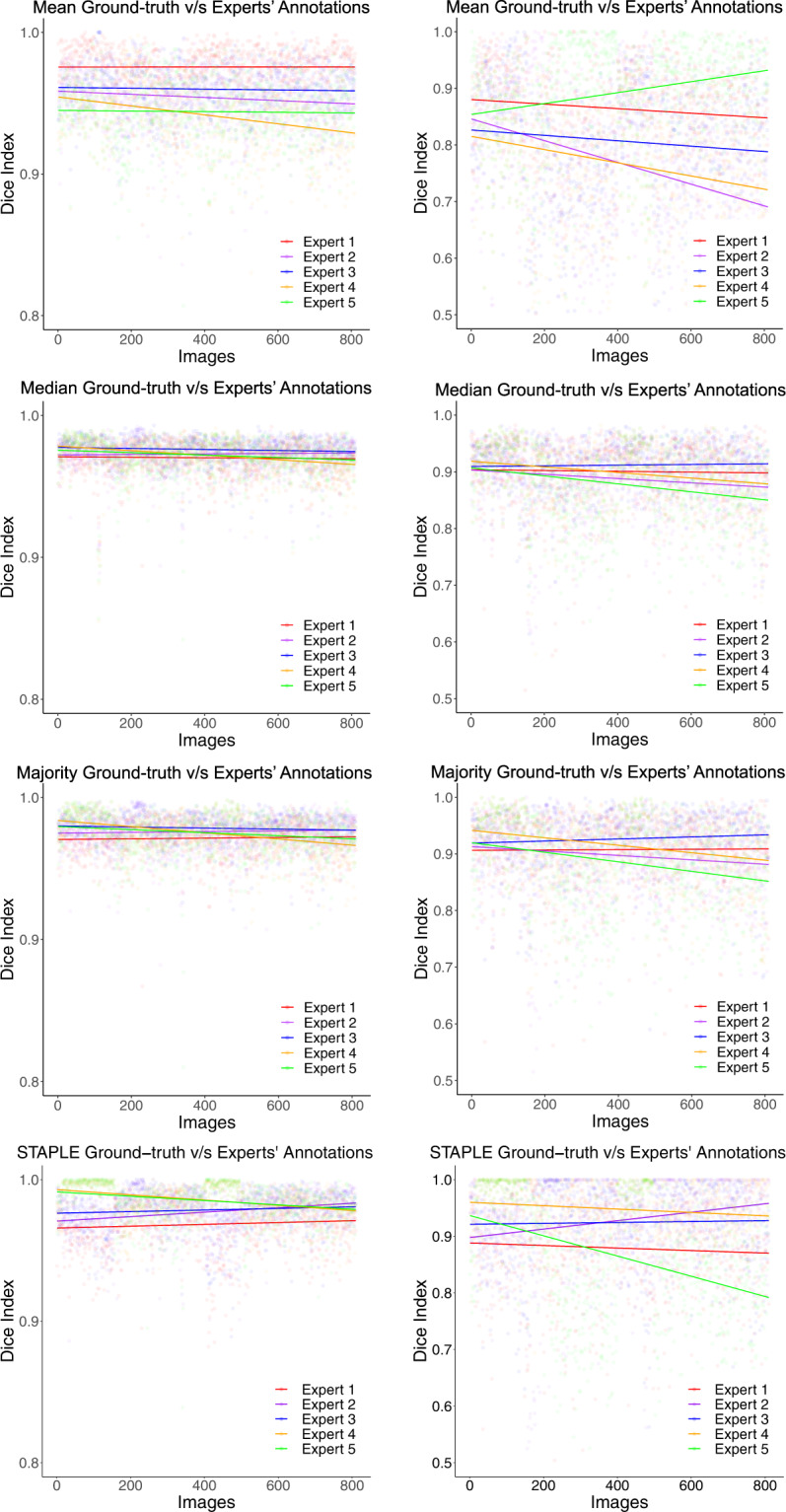
Comparison of robust linear regression plots for Dice index. Column 1: optic disc; Column 2: optic cup.

Expert-wise OD and OC segmentation variability analysis with respect to the mean, median, majority, and STAPLE based fusion is presented in Tables 3, 4, respectively, using the aforementioned performance indices. The best agreement between the experts’ OD and OC annotations is with the STAPLE algorithm, followed by the majority ground-truth, and median ground-truth. The performance indices are the least for mean ground truth. To train deep learning algorithms with data augmentation, the median ground-truth could also be considered as it is close to the STAPLE and majority ground-truths. Figure 6 shows a scatter plot of the Dice index for the 810 images under consideration. It is evident from the robust regression plots for Dice index in Fig. 6 that the experts’ annotations are close to STAPLE, majority, and median ground-truths rather than the mean ground-truth.

**Table 3:** Performance comparison of experts’ OD segmentation vs. mean, median, majority, and STAPLE OD ground-truth. The best performance indices overall are for the STAPLE algorithm, followed by the majority, median, and mean ground-truths.

Experts’ annotation vs. Mean ground-truth						
	*S*_*e*_	*S*_*p*_	*A*_*c*_	*E*_*p*_	*J*	*D*
Expert 1	0.9439	1.0000	0.9991	0.0009	0.9439	0.9696
Expert 2	0.9050	1.0000	0.9983	0.0017	0.9050	0.9486
Expert 3	0.9208	1.0000	0.9986	0.0014	0.9208	0.9583
Expert 4	0.8838	1.0000	0.9978	0.0022	0.8838	0.9365
Expert 5	0.8869	1.0000	0.9979	0.0021	0.8869	0.9383
**Average**	**0.9081**	**1.0000**	**0.9983**	**0.0017**	**0.9081**	**0.9503**
Experts’ annotation vs. Median ground-truth						
Expert 1	0.9878	0.9404	0.9989	0.0011	0.9404	0.9692
Expert 2	0.9689	1.0000	0.9989	0.0011	0.9440	0.9709
Expert 3	0.9794	0.9995	0.9989	0.0011	0.9472	0.9718
Expert 4	0.9571	0.9998	0.9989	0.0011	0.9431	0.9706
Expert 5	0.9586	0.9998	0.9989	0.0011	0.9428	0.9704
**Average**	**0.9704**	**0.9879**	**0.9989**	**0.0011**	**0.9435**	**0.9706**
Experts’ annotation vs. Majority ground-truth						
Expert 1	0.9902	0.9991	0.9989	0.0011	0.9425	0.9702
Expert 2	0.9735	1.0000	0.9990	0.0010	0.9503	0.9742
Expert 3	0.9825	0.9995	0.9991	0.0009	0.9513	0.9737
Expert 4	0.9617	0.9999	0.9990	0.0010	0.9496	0.9740
Expert 5	0.9633	0.9998	0.9990	0.0010	0.9494	0.9739
**Average**	**0.9742**	**0.9997**	**0.9990**	**0.0010**	**0.9486**	**0.9732**
Experts’ annotation vs. STAPLE ground-truth						
Expert 1	0.9976	0.9989	0.9989	0.0010	0.9397	0.9686
Expert 2	0.9875	0.9994	0.9991	0.0008	0.9550	0.9763
Expert 3	0.9939	0.9992	0.9991	0.0008	0.9535	0.9746
Expert 4	0.9840	0.9996	0.9993	0.0006	0.9628	0.9809
Expert 5	0.9843	0.9997	0.9993	0.0006	0.9652	0.9820
**Average**	**0.9894**	**0.9993**	**0.9991**	**0.0007**	**0.9552**	**0.9764**

**Table 4:** Performance comparison of experts’ OC segmentation vs. mean, median, majority, and STAPLE OC ground-truth. The performance indices are the best for the STAPLE algorithm, followed by majority, median, and mean ground-truths.

Experts’ annotation vs. Mean ground-truth						
	*S*_*e*_	*S*_*p*_	*A*_*c*_	*E*_*p*_	*J*	*D*
Expert 1	0.7556	1.0000	0.9988	0.0012	0.7556	0.8537
Expert 2	0.6202	1.0000	0.9977	0.0023	0.6202	0.7507
Expert 3	0.6709	1.0000	0.9983	0.0017	0.6709	0.7919
Expert 4	0.6100	1.0000	0.9977	0.0023	0.6100	0.7446
Expert 5	0.7890	1.0000	0.9990	0.0010	0.7890	0.8714
**Average**	**0.6891**	**1.0000**	**0.9983**	**0.0017**	**0.6891**	**0.8025**
Experts’ annotation vs. Median ground-truth						
Expert 1	0.9450	0.9994	0.9990	0.0010	0.8092	0.8906
Expert 2	0.8407	0.9998	0.9987	0.0013	0.7894	0.8787
Expert 3	0.9014	0.9997	0.9990	0.0010	0.8328	0.9067
Expert 4	0.8466	0.9999	0.9989	0.0011	0.8112	0.8932
Expert 5	0.9460	0.9992	0.9989	0.0011	0.7803	0.8730
**Average**	**0.8959**	**0.9996**	**0.9989**	**0.0011**	**0.8046**	**0.8884**
Experts’ annotation vs. Majority ground-truth						
Expert 1	0.9561	0.9993	0.9991	0.0009	0.8184	0.8950
Expert 2	0.8546	0.9998	0.9988	0.0012	0.8039	0.8866
Expert 3	0.9284	0.9996	0.9992	0.0008	0.8557	0.9196
Expert 4	0.8672	0.9999	0.9991	0.0009	0.8376	0.9086
Expert 5	0.9589	0.9992	0.9990	0.0010	0.7931	0.8792
**Average**	**0.9130**	**0.9996**	**0.9990**	**0.0010**	**0.8217**	**0.8978**
Experts’ annotation vs. STAPLE ground-truth						
Expert 1	0.9900	0.9988	0.9987	0.0012	0.7782	0.8666
Expert 2	0.9545	0.9995	0.9992	0.0007	0.8742	0.9280
Expert 3	0.9822	0.9991	0.9990	0.0009	0.8313	0.9015
Expert 4	0.9591	0.9995	0.9993	0.0006	0.8853	0.9364
Expert 5	0.9957	0.9985	0.9985	0.0014	0.7460	0.8450
**Average**	**0.9763**	**0.9990**	**0.9989**	**0.0009**	**0.8230**	**0.8955**

**Fig. 7:**
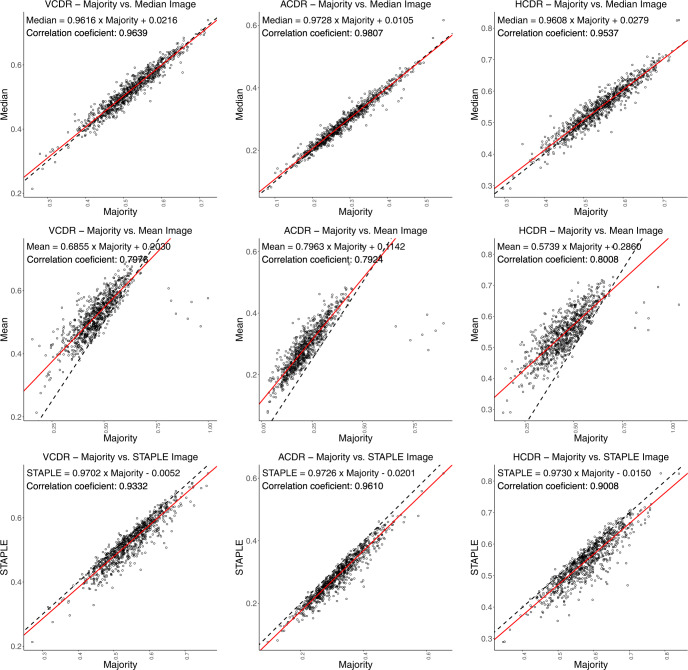
Robust linear regression analysis and intraclass correlation coefficient (Plots Set - 1). The 45° line is shown in dashed black line-style and the robust linear fit, using Huber’s method^38^, is shown in solid red line-style.

**Fig. 8:**
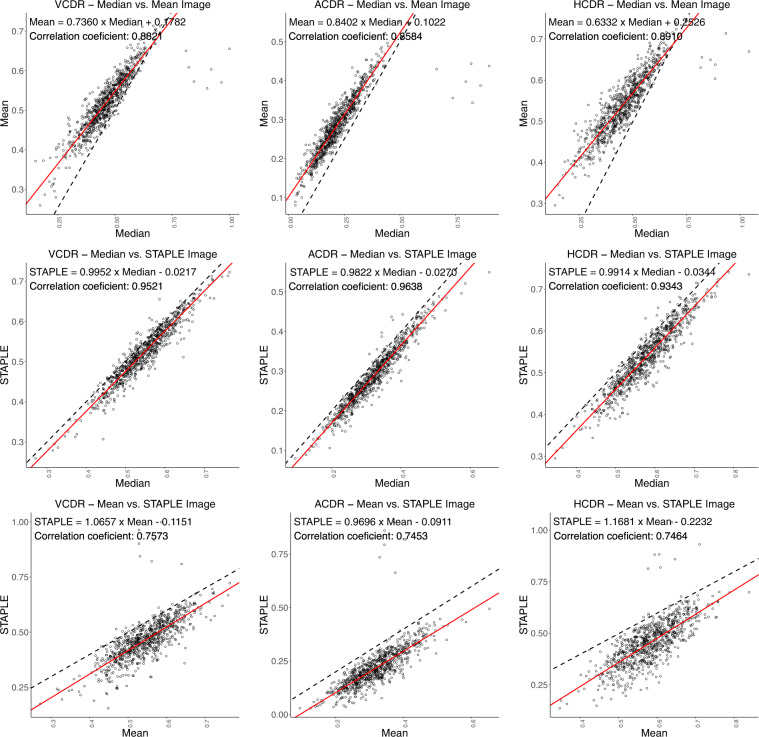
Robust linear regression analysis and intraclass correlation coefficient (Plots Set - 2). The 45° line is shown in dashed black line-style and the robust linear fit, using Huber’s method^38^, is shown in solid red line-style.

We present VCDR, HCDR, and ACDR ground-truth computed (cf. Fig. 1) from the various ground-truths. We perform a robust regression study with intraclass correlation coefficient (ICC) measurement (cf. Figs. 7, 8). Linear least-squares regression is easily affected by outliers and hence, in order to obtain a robust fit, we employed robust linear regression using the Huber loss function^38^. The ICC is a measure of reliability of measurements with 95% confidence interval. The closer the ICC is to +1.0 or −1.0, the greater the strength of the linear relationship between two methods for the same measurement. We found a strong linear relationship between ACDR–majority and median with ICC of 0.9807, followed by VCDR–majority and median with ICC of 0.9639 and weak linear relationship between ACDR–mean and STAPLE with ICC of 0.7453. The ICC quantifies the degree to which two methods are related.

**Fig. 9:**
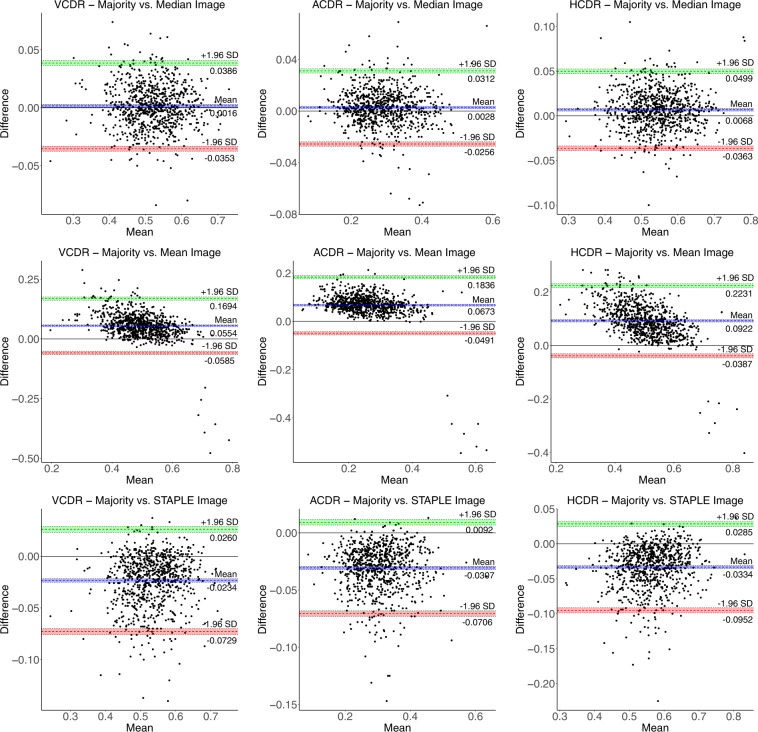
Bland-Altman plots (Set - 1) for VCDR, HCDR, and ACDR computed from mean, median, majority, and STAPLE ground-truths with limits of agreement ± 1.96 SD (standard deviation). The coloured shaded areas represent confidence interval limits for mean (blue) and agreement limits (green and red).

**Fig. 10:**
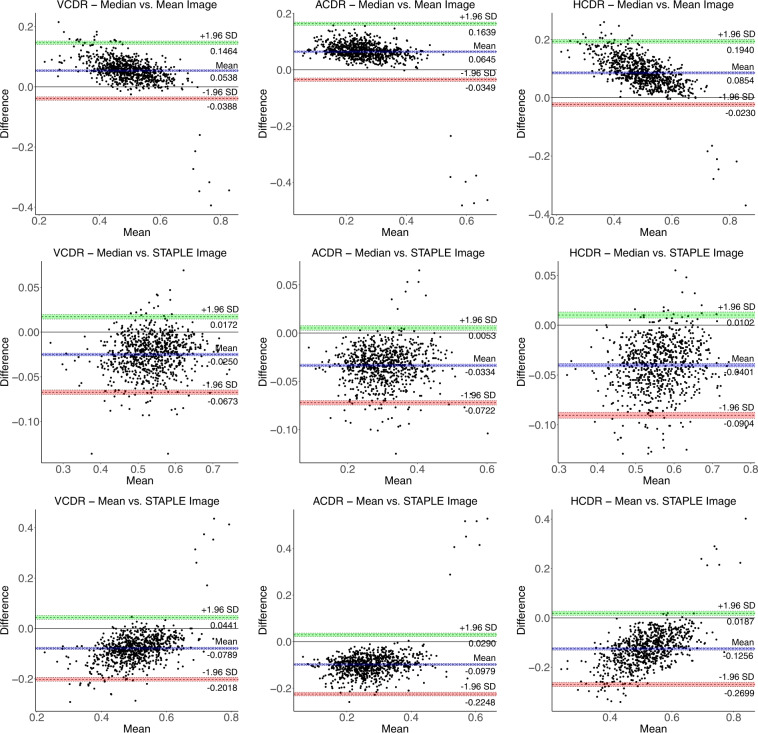
Bland-Altman plots (Set - 2) for VCDR, HCDR, and ACDR computed from mean, median, majority, and STAPLE ground-truths with limits of agreement ± 1.96 SD (standard deviation). The coloured shaded areas represent confidence interval limits for mean (blue) and agreement limits (green and red).

We use Bland-Altman plots (cf. Figs. 9, 10) to quantify the agreement between different ground-truths for the measurement of VCDR, HCDR, and ACDR. The Bland-Altman plot is a graphical way of comparing two measurement methods. It is a plot of the differences between the two methods against their average. Horizontal lines (in colour) are drawn at the mean difference (bias), and at the limits of agreement (LoA), which is defined as the mean difference ± 1.96 times the standard deviation (SD) of the differences. We observed a minimum bias of 0.0016 and 0.0028 for VCDR–majority vs. median and ACDR–majority vs. median, respectively, and a maximum bias of 0.1256 for HCDR–mean vs. STAPLE. The Bland-Altman plots show less than 5% of the 810 images as outliers for not being in the respective LoA. The majority and median Bland-Altman plots show the best agreement with minimum bias and narrower LoA.

**Fig. 11:**
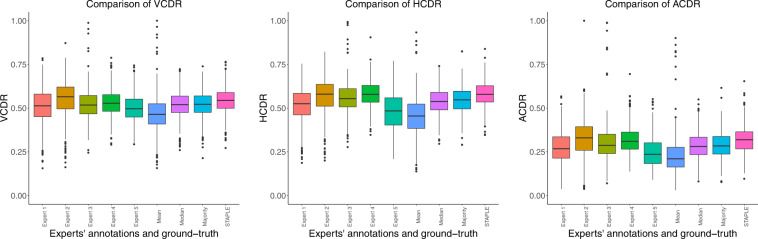
Adjusted box plots showing the distribution of VCDR, HCDR, and ACDR computed from the individual expert’s annotations and those computed from the mean, median, majority, and STAPLE ground-truths. The adjusted/robust box plots were generated using litteR package^39^.

Figure 11 shows *adjusted* box plots that depict the distribution of VCDR, HCDR, and ACDR computed from the experts’ annotations and various ground-truths. The adjusted box plots are more robust than the standard box plots as they account for skew and outliers in the data distribution^39^. The adjusted box plots of VCDR, HCDR, and ACDR computed using the STAPLE algorithm ground-truth have a large overlap with those computed from the outlines of several experts. This is closely followed by the majority and median ground-truth based VCDR, HCDR, and ACDR.

## Data Availability

No custom code was used.
