# Author Correction: *Chákṣu:* A glaucoma specific fundus image database

**DOI:** 10.1038/s41597-023-02084-4

**Published:** 2023-04-06

**Authors:** J. R. Harish Kumar, Chandra Sekhar Seelamantula, J. H. Gagan, Yogish S. Kamath, Neetha I. R. Kuzhuppilly, U. Vivekanand, Preeti Gupta, Shilpa Patil

**Affiliations:** 1 Department of Electrical and Electronics Engineering, Manipal Institute of Technology Manipal, Manipal Academy of Higher Education, Manipal, 576104 India; 2 Department of Electrical Engineering, Indian Institute of Science, Bangalore, 560012 India; 3 Department of Ophthalmology, Kasturba Medical College Manipal, Manipal Academy of Higher Education, Manipal, 576104 India; 4 Department of Ophthalmology, ASRAM, Eluru, 534005 India; 5 Hind Institute of Medical Sciences, Sitapur, 261303 India; 6 Aastha Superspeciality Eye Hospital, Banashankari, Bangalore, 560085 India

**Keywords:** Optic nerve diseases, Eye abnormalities, Optic nerve diseases, Eye abnormalities

Correction to: *Scientific Data* 10.1038/s41597-023-01943-4, Published online 03 February 2023

In this article the affiliation details for Preeti Gupta were incorrectly given as Department of Ophthalmology, Dr. Ram Manohar Lohia Institute of Medical Sciences, Lucknow, 226010, India but should have been Hind Institute of Medical Sciences, Sitapur, 261303, India. The original article has been corrected.

